# HOLLOW: Generating Accurate Representations of Channel and Interior Surfaces in Molecular Structures

**DOI:** 10.1186/1472-6807-8-49

**Published:** 2008-11-14

**Authors:** Bosco K Ho, Franz Gruswitz

**Affiliations:** 1 Department of Biochemistry and Biophysics, Howard Hughes Medical Institute, University of California, San Francisco, San Francisco CA 94158-2517, USA; 2 Department of Biochemistry and Biophysics, Center for Structure of Membrane Proteins (CSMP), University of California, San Francisco, San Francisco CA 94158-2517, USA

## Abstract

**Background:**

An accurate rendering of interior surfaces can facilitate the analysis of mechanisms at atomic-level detail, such as the transport of substrates in the ammonia channel. In molecular viewers, one must remove the exterior surface that obscures the channel surface by clipping the viewing plane or manually selecting the channel residues in order to display a partial surface. Neither method is entirely satisfactory, as unwanted additional pieces of surfaces are always generated.

**Results:**

To cleanly visualize a channel surface, we present HOLLOW, a program that generates a "casting" of the interior volume of the protein as dummy atoms. We show that the molecular surface of the dummy atoms closely approximates the channel surface, where this complementary surface of the protein channel can be displayed without superfluous surfaces.

**Conclusion:**

The use of HOLLOW significantly simplifies the generation of channel surfaces, and other interior surfaces of protein structures. HOLLOW is written in PYTHON and is available at .

## Background

The surface of a protein can be used to rationalize complex biochemical mechanisms. For instance, in studies of protein channels, the geometric properties of the channel openings dictate the type of substrates that can pass through the channel. Vestibules in the middle of channels can serve as staging areas of the substrate transport. Programs that identify trajectories in a protein channel (HOLE [1], CAVER [2], and MOLE [3]) can generate representations of the channel surface, but these surfaces are rendered at a coarse level of detail. In order to fully realize an analysis of the channel, an atomic-level visualization of the channel surface is required.

Unfortunately, in standard molecular viewers, extraneous pieces of surfaces are a persistent problem in the selective representation of molecular surfaces. The reason is that partial surfaces are, by convention, encoded as a property of an atom. Certain atoms in a protein structure may contribute to more than one surface where for example, an atom lining a channel may also form part of a void. If the surface of this atom is selected to be displayed, then both the channel and void surfaces will be displayed, where the partial surface of the void is an artifact if the area of interest is the channel.

Another common problem is the selection of channel residues. As the exterior part of the molecular surface obscures the channel surface, the exterior surface must also be removed. Typically, the viewing plane is clipped and the surface representation is restricted to the display of channel-lining residues. Unfortunately, there is no simple way to select channel residues and the channel residues must be selected manually, which is a tedious and error-prone process for the user.

To overcome these problems, we present HOLLOW, a program that produces output that can be used to generate clean visualizations of channel surfaces in a standard molecular viewer, such as PyMOL [4]. The output of HOLLOW is a PDB file of dummy atoms that makes a "casting" of the empty volumes of a structure where the molecular surface of the dummy atoms can be shown to closely approximate the channel surface. The channel residues can also be selected by proximity to the dummy atoms. The display of this complementary surface of the dummy atoms results in a clean visualization of the channel without any unwanted additional surfaces.

## Implementation

HOLLOW uses dummy atoms to fill the empty space inside a structure. In the automated mode, overlapping dummy atoms are defined on a rectangular grid surrounding the molecular structure. By default, the probe radius of the dummy atoms is set to 1.4 Å and the grid-spacing to 0.5 Å. The dummy atoms are eliminated from the grid if they overlap with the molecular structure, or they are found outside the exterior envelope of the protein. To reduce the number of dummy atoms while not affecting the overall volume, dummy atoms from the interior of the block are selectively removed if this does not affect the overall volume of the block. These dummy atoms are then written to a PDB file as oxygen atoms by default, or any other atom type as specified in the configuration file. These dummy atoms constitute a global map of voids, pockets and tunnels of a protein structure.

In order to identify pockets and tunnels, an exterior envelope needs to be defined. As channels open from multiple positions of the protein, the exterior envelope is used to demarcate the end of the channel. Dummy atoms found above the envelope are eliminated. The dummy atoms below the envelope and above the protein surface correspond to the volume of pockets. In HOLLOW, the exterior envelope is defined by rolling a large 8.0 Å sphere over the surface atoms. Surface atoms are defined by the solvent-accessible surface area (SASA) by SASA > 9 Å^2^. Both the SASA and exterior envelope elimination are calculated using the Shrake-Rupley dot-density method [5].

HOLLOW allows for a wide range of customization options. All parameters, including grid spacing, probe sizes and fill atom type, are editable. In particular, there is a manual mode where the user can specify a constrained region (spherical or cylindrical) in which to calculate the dummy atoms. This allows finer grid spacing to be used with reasonable processor time.

## Discussion

**Figure 1:**
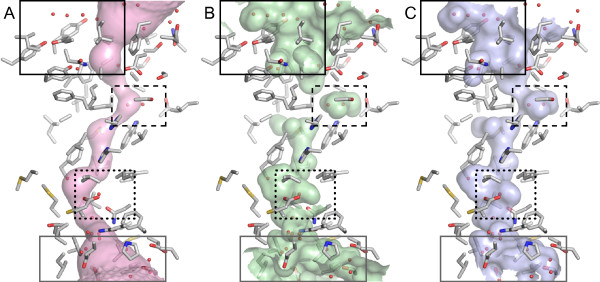
**Representations of channel surfaces**. Comparison of (A) MOLE tunnel, (B) selected molecular surface, (C) and HOLLOW surface. The periplasmic opening (black box) and the cytoplasmic opening (grey box) regions show a clear loss of detail in the MOLE tunnel and superfluous detail in the selected molecular surface. The "gating" region of the periplasmic vestibule (dashed box) and the cytoplasmic vestibule (dotted box) exhibit comparable detail in the selected surface and HOLLOW surface, while the MOLE tunnel lacks the details necessary for the representation of the channel lumen.

To illustrate the improvements in the visualization of channels surfaces with HOLLOW, we use the example of the ammonia channel Rh50 of *Nitrosomonas europaea *(PDB ID 3BHS) [6]. One commonly available method is to generate a channel trajectory with the program MOLE [3]. The MOLE-generated surface corresponds to a spherical volume from the center of the channel at each point along the path and illustrates the two most likely average paths of ammonia conduction (Figure 1A). However, the MOLE-generated surface lacks atomic details: the surface does not suggest a mechanism for "gating" (dashed box), nor identify vestibules for the substrate (dotted box), nor illustrate the entire apertures to the channel (solid boxes).

By running HOLLOW on Rh50, dummy atoms (defined on a 0.5 Å grid) are generated to fill the channel volume. The channel residues are easily selected by proximity to the dummy atoms. The display of the molecular surface can be restricted to the surface of the channel residues, which produces a highly detailed visualization of the channel surface in atomic detail (Figure 1B). Nevertheless, there are unnecessary additional surfaces in this display (Figure 1B – left side and lower right of black solid box, lower right of dotted box, and grey box), which arise from the display of other surfaces associated with the atoms in the channel residues. In comparison, the molecular surface of the HOLLOW-generated dummy atoms is shown (Figure 1C), which clearly shows the channel lumen in atomic detail without gratuitous surfaces. Comparison to the molecular surface of the channel (atoms selected by proximity to the HOLLOW-generated dummy atoms – Figure 1B) reveals a very similar surface with only minor differences.

**Figure 2:**
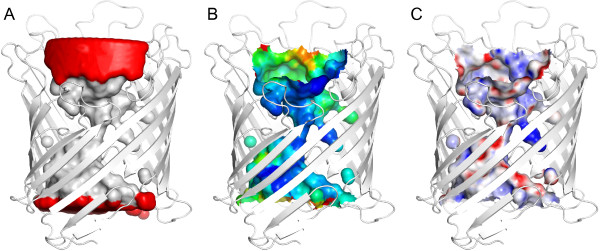
**Surface properties on HOLLOW surface**. (A) The opening of a surface channel can be specified in terms of a cylindrical constraint by displaying the surface of the dummy atoms found wholly inside the constraint (white) while hiding the surface of dummy atoms on the surface of the constraint (red). (B) The B-factor of the HOLLOW surface can be depicted by color to indicate accuracy of the molecular surface. (C) The mapping of electrostatic potential onto the HOLLOW surface can also be achieved in PyMOL with the APBS plug-in [11,12].

The use of HOLLOW-generated dummy atoms also permits control over other aspects of surface visualization. The displayed surface can be customized by the selection of a subset of dummy atoms. It is important to note that only part of the molecular surface of a group of dummy atoms is complementary to the protein surface. However, the selective display of the complementary surface can be easily controlled using HOLLOW in the manual mode with constraints. For example when a cylindrical constraint is used to generate a "casting" of the protein channel, dummy atoms found on the surface of the cylindrical constraint are tagged with occupancy = 0 while those inside are tagged with occupancy = 1 (Figure 2A). By restricting the surface to dummy atoms with occupancy = 1, a channel surface is displayed where the opening is defined by the intersection of the cylindrical constraint to the molecular surface of the protein (Figure 2B). As HOLLOW stores the average B-factor of nearby protein atoms to the B-factor of a dummy atom, the accuracy of the channel surface can be illustrated by coloring the HOLLOW-generated surface to this B-factor (Figure 2B). Similarly, the electrostatic potential of the heavy atoms near each dummy atom can be assigned to the surface resulting in an electrostatic potential map of the channel surface (Figure 2C).

Compared to other surface generation programs, HOLLOW focuses on customization of surface rendering. VOIDOO [7] and SURFNET [8] generate polygon surfaces, which cannot be easily edited in molecular viewers. Instead, HOLLOW generates dummy atoms, which not only provides a convenient tool for selecting residues that line cavities by proximity to the dummy atoms, but also permits control over the rendering of partial surfaces leading to significantly clearer visualizations of channel surfaces. Indeed, the origin of HOLLOW arose from the difficulty that the authors found in generating detailed channel surfaces using standard existing programs.

**Figure 3:**
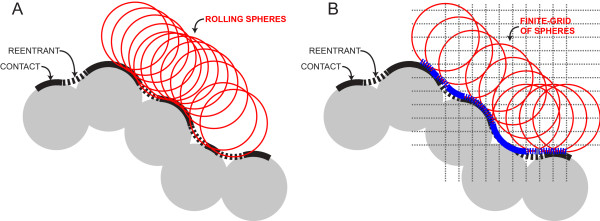
**Molecular surface grid-approximation**. (A) The molecular surface (black) is defined by the atomic contact of a rolling sphere and the reentrants (dashed) resulting at the sphere contacts of multiple atoms. (B) The finite-grid approximation in HOLLOW results in a surface (blue) that closely approximates the molecular surface.

The van der Waals (vdW) surface of the HOLLOW-generated dummy atoms at an infinitely-small grid-spacing can be shown to be an exact representation of the molecular surface of the protein. The molecular surface of the protein is commonly defined in terms of the Connolly surface [9], which is a smoothly differentiable surface defined over the surface of the protein. As the vdW surface of the protein contains abrupt cusps between overlapping atoms, the Connolly surface defines reentrant surfaces over the cusps (Figure 3A). The reentrant surface is defined as the surface traced out by a probe sphere rolling over the protein. The vdW surface of the dummy atoms, as the grid-spacing approaches zero, approaches the surface of a probe sphere rolling over the protein surface, which is the Connolly surface (Figure 3B).

**Figure 4:**
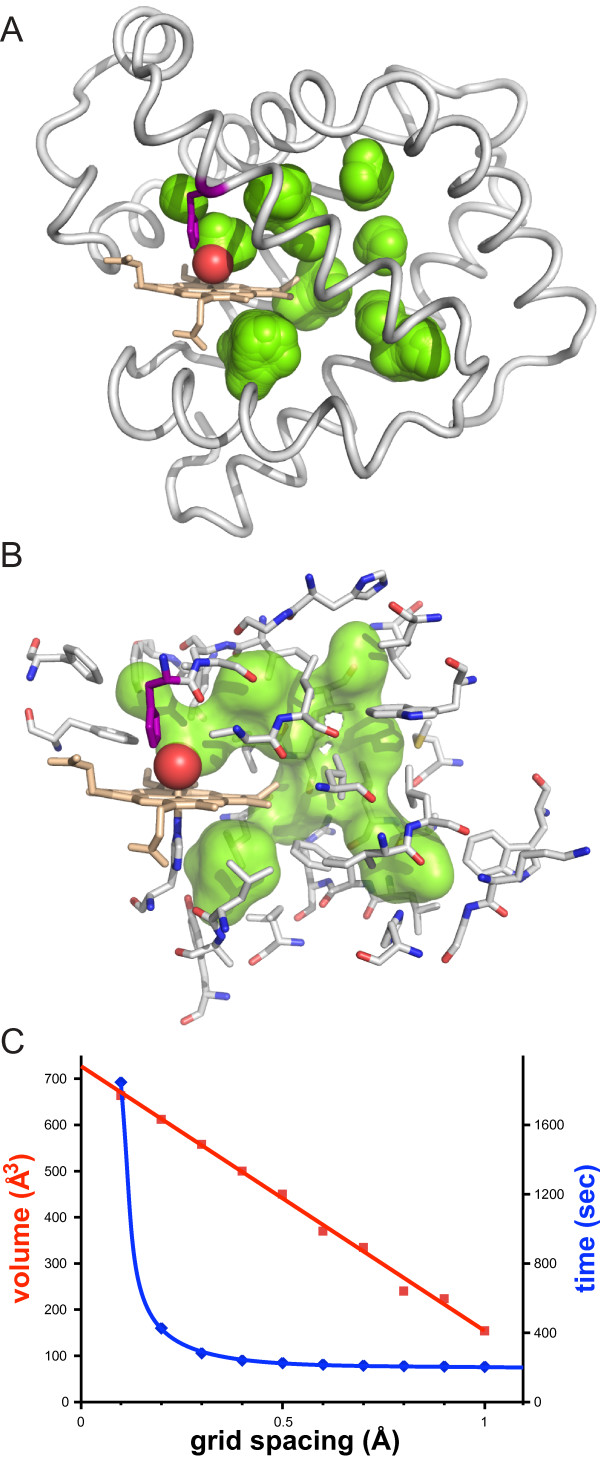
**The interior volume of myoglobin**. (A) The voids (green) identified in myoglobin using a grid-spacing of 0.2 Å. (B) With the volume-filling spheres, it is easy to select the residues (stick display) that define the interior volume by proximity to the volume-filling spheres. The interior volume is shown as a semi-transparent surface. (C) At increasingly small spacing the finite-grid approximation approaches ideality as seen in the convergence of the volume (red) of the dummy atoms for the voids in myoglobin. The time required to generate the dummy atoms at different grid-spacing (blue) shows a significant jump in time from a grid-spacing of 0.2 Å to 0.1 Å. The calculations were performed on an iMac where the optional compilation module Psyco [13] was used to accelerate the calculation.

To determine the accuracy of the HOLLOW-generated spheres as a function of grid spacing, one could measure surface area or volume of a set of dummy atoms. Though a good measure for the representation of a surface, closely overlapping spheres using the Shrake-Rupley dot-density method require an unreasonably high number of dots for an accurate surface area. In contrast, a grid-based volume approximation of the overlapping spheres generates an accurate value for the volume with a reasonably coarse grid spacing. As an example, interior volumes of myoglobin [10] (PDB ID 1J52) (Figure 4A), which form a fully-contained network of interior pathways (Figure 4B), can be used to assess the accuracy of the HOLLOW sphere approximation (Figure 4C) (details available on the website). As the grid spacing decreases, the volume of the dummy atoms increases linearly to an asymptotic value of ~720 Å^3^, corresponding to the ideal volume as defined by the Connolly surface. At a grid-spacing of 0.5 Å, the volume of the dummy atoms constitutes 70% of the ideal volume, providing a reasonable approximation to the ideal volume. For higher accuracy, a grid-spacing of 0.1 Å results in dummy atoms that constitute 93% of the ideal volume. The calculation time increases exponentially with grid-spacing and grid volume, however even at grid spacing of 0.2 Å and a 27000 Å^3 ^grid, results can be obtained in minutes on most desktop computers.

## Conclusion

HOLLOW takes a "casting" of the cavities by filling the voids, channels and pockets of a protein with dummy atoms defined on a grid. At small grid-spacing, the surface of the casting is shown to match the molecular surface of the protein. Manipulation of the dummy atoms provides a convenient way to customize the visualization of the interior surface. The size of the grid and search region can dramatically affect the calculation time, but high quality surfaces can be determined in reasonable durations on average desktop computers.

## Availability and requirements

• Project name: Hollow

• Project home page: 

• Operating system(s): Platform independent

• Programming language: Python

• License: GNU GPL

## Authors' contributions

FG conceived the original idea. Both authors designed the program. BKH implemented the program. BKH and FG drafted the manuscript.
